# Odevixibat treatment in a child with hypoplastic left heart syndrome and severe cholestatic pruritus: a case report

**DOI:** 10.3389/fped.2024.1443338

**Published:** 2025-01-23

**Authors:** Rainer Ganschow, Christof Maucksch, Peter Rauschkolb, Martin B. E. Schneider

**Affiliations:** ^1^ Department of Pediatrics, University Children’s Hospital, Bonn, Germany; ^2^ Global Medical Affairs, Ipsen Pharma GmBH, Munich, Germany; ^3^ Department of Cardiology, Pediatric Heart Center, University Hospital of Bonn, Bonn, Germany

**Keywords:** odevixibat, cholestasis, hypoplastic left heart syndrome, pruritus, case report

## Abstract

Liver-related abnormalities are commonly observed in patients with congenital heart disease, and these may lead to secondary manifestations such as pruritus. Odevixibat is an ileal bile acid transporter inhibitor under investigation for the treatment of cholestatic liver diseases. Here, we describe the effects of odevixibat treatment in a pediatric patient with congenital heart disease and severe cholestatic pruritus. A 2-year-old male with Kleefstra syndrome, hypoplastic left heart syndrome, and a history of Giessen procedure and biventricular correction surgery presented to the pediatric cardiology and hepatology outpatient clinics at University Children's Hospital Bonn. Portal hypertension was evident on imaging, and the patient was experiencing severe itching attacks that did not respond to treatment with naltrexone, ursodeoxycholic acid, dimetindene, or rifampicin. Sleep and quality of life were poor. Treatment with odevixibat was initiated off label due to refractory pruritus and elevated serum bile acids. Improvements in pruritus and sleep occurred rapidly with odevixibat and were sustained for the duration of treatment. The patient's serum bile acids decreased from 111 μmol/L before treatment with odevixibat to 24 μmol/L within 1 month of initiating therapy. Relief from pruritus had positive effects on psychomotor development and quality of life. Mild diarrhea lasting 2 days was reported by the patient's mother. In this case report, odevixibat was effective and well tolerated. Together with those of previous studies in patients with progressive familial intrahepatic cholestasis and Alagille syndrome, these results suggest that odevixibat warrants further study as a potential treatment option for patients with cholestatic pruritus of diverse etiologies.

## 1 Introduction

It is well established that patients with congenital heart disease can develop liver abnormalities and secondary hepatic disease, known as cardiac hepatopathy, as a result of passive venous congestion and/or reduced cardiac output, both in those who have undergone Fontan palliative surgery and in those who have not (1–7). For example, among patients with hypoplastic left heart syndrome [HLHS; a spectrum of cardiac malformations characterized by underdevelopment of the left heart and aorta (8)], a retrospective autopsy study found that 43% (18/42) also had hepatic necrosis (9).

Signs of cardiac hepatopathy can include cholestasis, fibrosis, and hepatocellular carcinoma (1, 10). Cholestasis is a condition of impaired bile flow commonly associated with pruritus, jaundice, and elevated levels of serum bile acids and liver enzymes (11, 12). Although there are some reports of pruritus in patients with heart failure (13, 14), little is known about the prevalence or treatment of cholestatic pruritus in patients with cardiac hepatopathy.

Odevixibat is a potent, selective inhibitor of the ileal bile acid transporter (IBAT). In phase 3 studies in patients with cholestatic liver disease (ie, progressive familial intrahepatic cholestasis (PFIC) and Alagille syndrome) and pruritus, odevixibat treatment significantly reduced symptoms of pruritus and serum bile acid levels (15–17). Based in part on data from these studies, odevixibat was approved by the European Medicines Agency for treatment of PFIC (18) and for treatment of cholestatic pruritus in Alagille syndrome (19) and by the United States Food and Drug Administration for treatment of pruritus in patients with PFIC and Alagille syndrome (20).

Here, we describe the effects of odevixibat treatment in a pediatric patient with HLHS and severe cholestatic pruritus. This patient was also diagnosed with Kleefstra syndrome, a rare genetic disorder associated with intellectual disability, hypotonia, distinct facial features, and occasionally, heart defects, including HLHS (21, 22).

## 2 Case report

In September 2021, a 2-year-old male with a diagnosis of HLHS presented to the pediatric cardiology outpatient clinic and subsequently to the pediatric hepatology outpatient clinic at University Children's Hospital Bonn for a second opinion on treatment options. The patient had been born in July 2019 at 36 weeks and 6 days of gestation, and serial echocardiograms in the week following birth revealed mitral and aortic stenosis and a hypoplastic aortic arch with ductus-dependent system perfusion. The patient underwent the Giessen hybrid procedure 5 days postnatally, followed by biventricular corrective surgery at 4 months of age. The patient's postoperative course was complicated by development of kidney failure and cholestasis. Magnetic resonance cholangiopancreatography showed cholangitis, liver fibrosis, significant splenomegaly, and portosystemic shunt via the umbilical veins, suggesting portal hypertension. Follow-up gastroscopy and ultrasound revealed 2nd-degree esophageal varices, which confirmed the diagnosis of portal hypertension, and abdominal ultrasound showed hepatofugal flow and hepatosplenomegaly (Table 1). There were no clinical or histological signs indicating typical cholestatic liver diseases such as PFIC or Alagille syndrome. The patient was also diagnosed with Kleefstra syndrome following a genetic test that identified a large deletion on chromosome 9q34.3. In addition to the genetic analysis for Kleefstra syndrome, a next-generation sequencing analysis was performed to exclude genetic nephrologic disease; however, no analysis of genes related to cholestasis was performed.

**Table 1:** Pre-odevixibat investigations in a patient with HLHS and severe cholestatic pruritus.

Investigation	Findings
Gastroscopy	HLHS (<5 mm diameter in the lower third of the esophagus); esophageal varices associated with portal hypertension
MRCP	Cholangitis, liver fibrosis, splenomegaly, portosystemic shunt via the umbilical veins
Biopsy	• No signs of hepatitis (ie, no “giant cells”) • Staining against alpha-1-antitrypsin was negative • Immunohistochemistry excluded PFIC syndromes (MDR3, BSEP, etc) • No bile duct proliferation was found
Ultrasound Imaging	Hepatosplenomegaly; hepatofugal flow; hydronephrosis of the left kidney
Clinical Presentation	Scratch marks covering the whole body^a^; jaundice; sleep disturbance; psychomotor developmental delay

^a^ Pruritus was caregiver and physician assessed (ie, no formal scale was used). BSEP, bile salt export pump; HLHS, hypoplastic left heart syndrome; MRCP, magnetic resonance cholangiopancreatography; MDR3, multidrug resistance protein 3; PFIC, progressive familial intrahepatic cholestasis.

At the time of presentation to University Children's Hospital Bonn, the patient was in good general condition. He showed no signs of heart failure in everyday life, with oxygen saturation values at 100% and no evidence of tachypnea or increased sweating. The patient's mother reported that he was experiencing severe itching attacks (ie, pruritus), and scratch marks were evident all over his body upon physical examination (Table 1). The patient had to constantly wear gloves to prevent scratching and bleeding, and nighttime sleep was greatly disrupted for both the patient and his parents. Microcephaly and significant psychomotor developmental delay were also observed in the patient.

The patient continued to suffer from severe itching and impaired quality of life at 3 years of age. His pruritus was refractory to conventional treatments including naltrexone, ursodeoxycholic acid, or dimetindene oral drops. Rifampicin was also trialed, but because the medication was not well tolerated and resulted in elevations of liver transaminases, it was stopped after 2 weeks. Serum bile acid and liver function test levels were persistently elevated (ie, levels were elevated several fold vs. normal limits across multiple visits) (Table 2). Oral odevixibat 40 µg/kg/day was prescribed off label in November 2022 for ongoing pruritus and elevated serum bile acids; during odevixibat treatment, the patient also received ursodeoxycholic acid (2 × 150 mg).

**Table 2:** Laboratory values over time in a patient with HLHS and severe cholestatic pruritus.

Laboratory test	Values pre-odevixibat, median (range)	Values post-odevixibat, median (range)	Reference range or normal limit
ALT (U/L)	89 (66–586)	77 (43–222)	7–29
AST (U/L)	101 (84–650)	82 (26–179)	19–71
GGT (U/L)	285 (71–3,345)	93 (36–108)	2–15
Total bilirubin (mg/dL)	2.1 (0.8–10.2)	1.7 (1.0–6.6)	1.4
Direct bilirubin (mg/dL)	1.6 (0.6–8.6)	3.6 (0.7–6.2)	0.3
INR	1 (1–1)	1	0.9–1.1
Serum bile acids (μmol/L)	111 (49–290)	37 (24–40)	<10
Platelets (×10^9^/L)	127 (111–176)	69 (63–91)	200–460

Reference ranges or normal limits were derived from central laboratory report.

ALT, alanine aminotransferase; AST, aspartate aminotransferase; GGT, gamma-glutamyl transferase; HLHS, hypoplastic left heart syndrome; INR, international normalized ratio.

**Figure 1:**
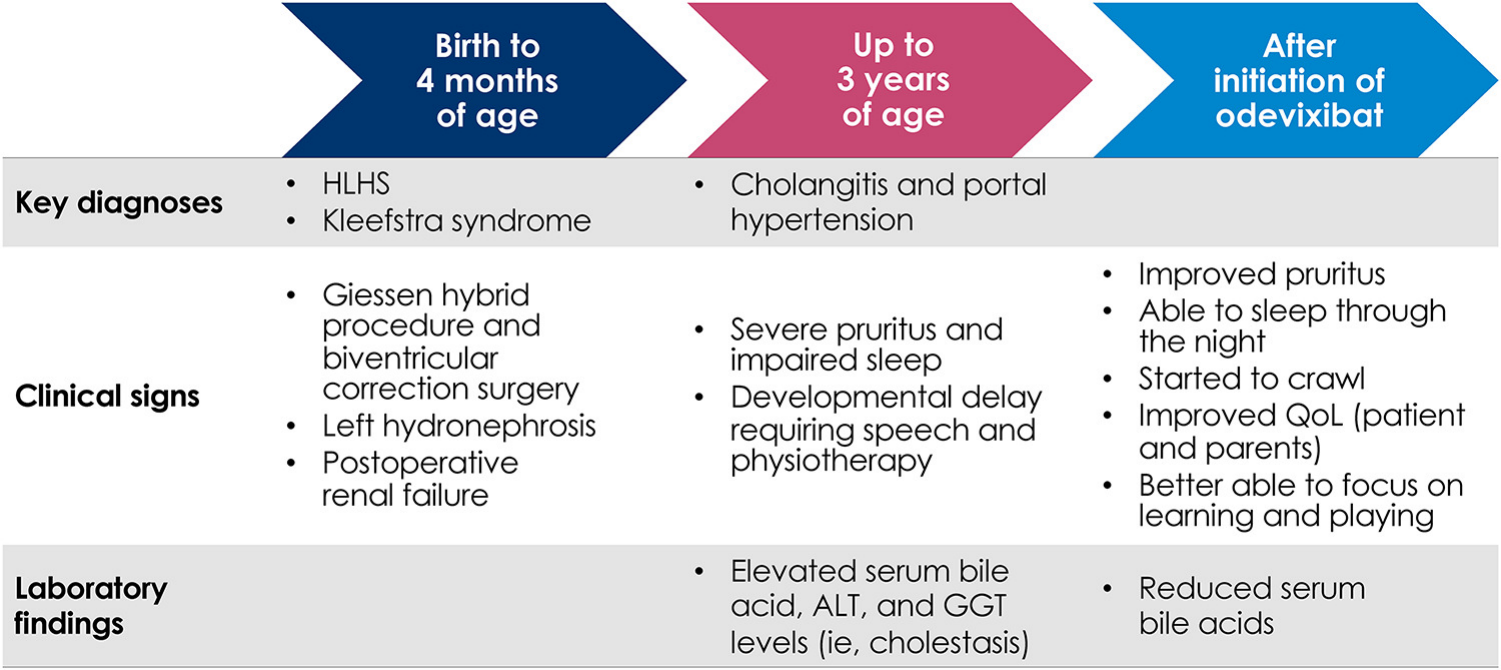
Flow diagram of key clinical milestones for patient with HLHS and severe cholestatic pruritus treated with odevixibat. ALT, alanine aminotransferase; GGT, gamma-glutamyl transferase; HLHS, hypoplastic left heart syndrome; QoL, quality of life.

Within 2 days of initiating odevixibat, marked improvements in pruritus and sleep were observed that were maintained over 6 months. The patient's pruritus was primarily observed by his parents and also by the clinician during physical exam. The patient was no longer scratching or bleeding and did not require gloves; subsequently, the patient's fine motor skills and psychomotor development progressed rapidly. He began to crawl and sleep through the night for the first time since infancy. Overall, the patient's mother reported his quality of life was greatly improved; he was happy, laughing, and better able to focus on learning and playing (Figure 1).

**Figure 2:**
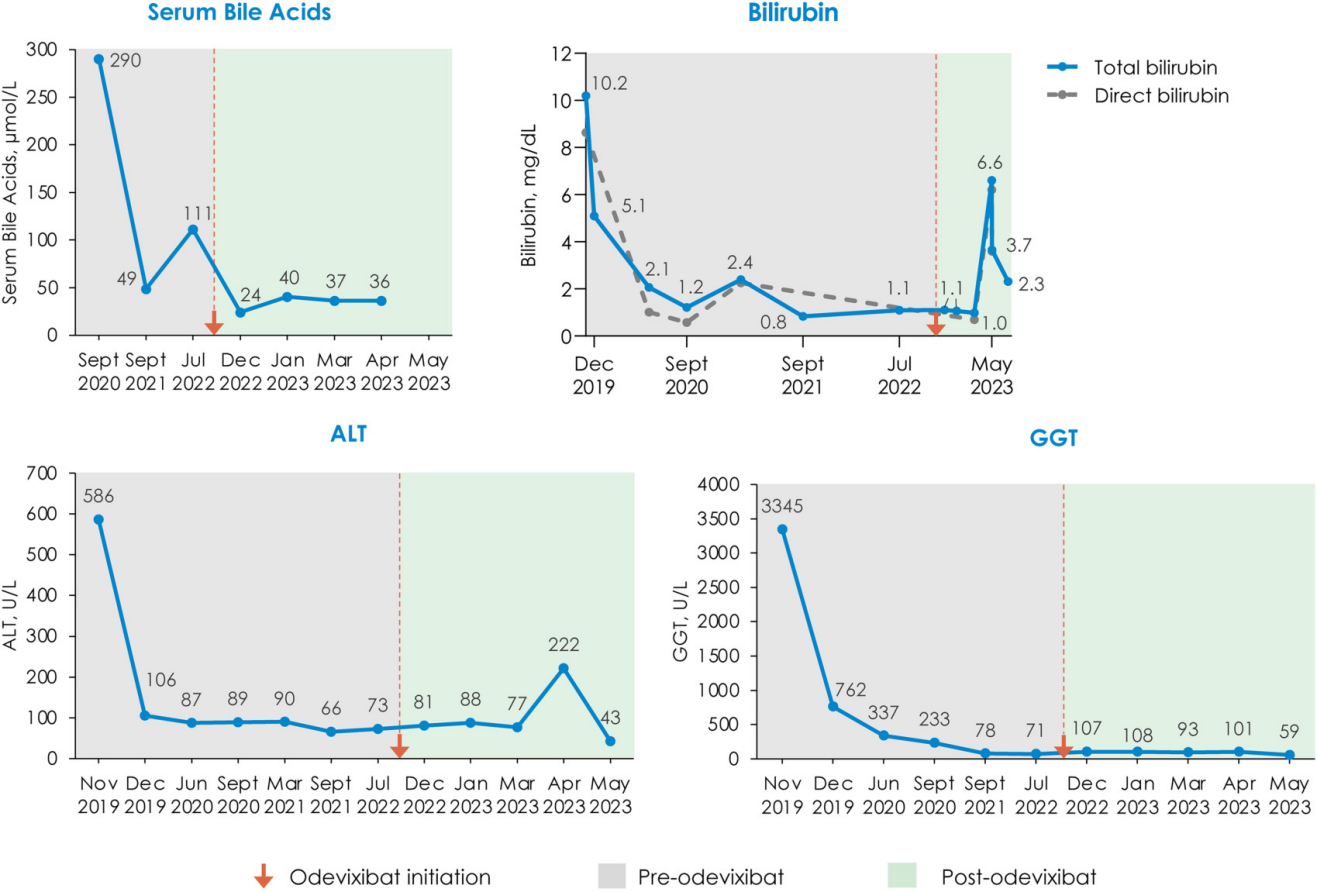
Changes in serum bile acids, bilirubin, ALT, and GGT with odevixibat treatment. All available laboratory values on a per-month basis are plotted. In May 2023, three laboratory assessments were taken within 4 days of each other; the last available value is plotted for ALT and GGT; for total bilirubin, all 3 May 2023 laboratory assessments are plotted. The bilirubin panel shows total bilirubin values with the solid line and direct bilirubin values with the dashed line; values for total bilirubin are labeled. ALT, alanine aminotransferase; GGT, gamma-glutamyl transferase.

Serum bile acids decreased from a pretreatment level of 111 μmol/L to 24 μmol/L within 1 month of initiating odevixibat; this reduction in serum bile acids was maintained at the last available assessment in April 2023 (36 μmol/L) (Figure 2). Total bilirubin and alanine aminotransferase (ALT) levels generally remained stable for the first 3 months after odevixibat initiation and then increased. The increase in ALT was transient; at the last available assessment in May 2023, the patient's ALT was lower than pre-odevixibat levels. The last available total bilirubin value in May 2023 was approximately 1.6-fold higher than the upper limit of normal; however, a specific clinical etiology for this perturbation was not apparent, and longer follow-up is not available as the patient passed away during a subsequent heart surgery.

Other than mild diarrhea lasting 2 days (reported by the patient's mother) post-odevixibat initiation, odevixibat was well tolerated in the patient.

A lay summary of the case details presented here can be found in the Supplementary Appendix.

## 3 Discussion

This case report is the first to demonstrate the sustained effectiveness of the IBAT inhibitor odevixibat in treating pruritus in a child with cardiac hepatopathy. While cardiac hepatopathy usually occurs many years after a Fontan procedure, the liver in patients with congenital heart disease may experience damage from the time of birth, and relevant liver fibrosis may be present in younger years (6, 7, 23–25). The patient described here with HLHS experienced chronic itching that led to significant impairments in sleep and quality of life. Following failure of other antipruritic agents, treatment with odevixibat rapidly and robustly improved the patient's pruritus, sleep, and quality of life and lowered serum bile acid levels.

This case also highlights the substantial burden of disease associated with chronic pruritus. Consistent with observations in patients with PFIC and Alagille syndrome (26–30), pruritus was among the most debilitating symptoms experienced by this patient and directly contributed to poor quality of life in both the patient and his family. The pruritus reductions with odevixibat were accompanied by improvements in the patient's mood, attention, and motor coordination (eg, ability to crawl and play).

Odevixibat was well tolerated in this patient, with diarrhea being the only side effect reported by the patient's mother. The diarrhea event was mild, transient, and consistent with results observed in previous studies of IBAT inhibitors [ie, odevixibat or maralixibat] (15, 17, 31, 32). Following initiation of odevixibat, there were some fluctuations in the patient's ALT and bilirubin values, which is consistent with previously reported findings from a phase 3 trial of odevixibat in patients with PFIC (17). In that study, while some patients generally had improvements in hepatic laboratory values with odevixibat, some mean values remained elevated (17).

In summary, odevixibat had rapid and sustained effects on itching, sleep, and serum bile acids in a patient with congenital heart disease and severe cholestatic pruritus. Together with those of previous studies in patients with PFIC and Alagille syndrome, these results suggest that odevixibat warrants further study as a potential treatment option for patients with cholestatic pruritus of diverse etiologies.

## Data Availability

The datasets presented in this article are not readily available to preserve the individual's privacy. Reasonable requests to access the datasets should be directed to the corresponding author.
