# Supplementary material for: Odevixibat treatment in a child with hypoplastic left heart syndrome and severe cholestatic pruritus: a case report
Source: Front Pediatr. 2025 Jan 23;12:1443338. doi: 10.3389/fped.2024.1443338 (PMC11799544; doi:10.3389/fped.2024.1443338)
Supplement: Supplementary file 1 [file Supplementaryfile1.docx]

Supplementary Appendix

*Lay Summary*

Some patients with heart problems also develop symptoms related to liver disease that cause a feeling of extremely itchy skin called pruritus. This article describes a 2-year-old boy with an underdeveloped heart (a condition known as hypoplastic left heart syndrome), as well as a genetic condition that affects development known as Kleefstra syndrome. The patient experienced itching so severe that he always wore gloves; it also caused major disruptions to his and his family’s everyday life. Once the patient started taking a new medication called odevixibat, he had profoundly less itch and better sleep, and as a result, he started laughing, playing more, and crawling.


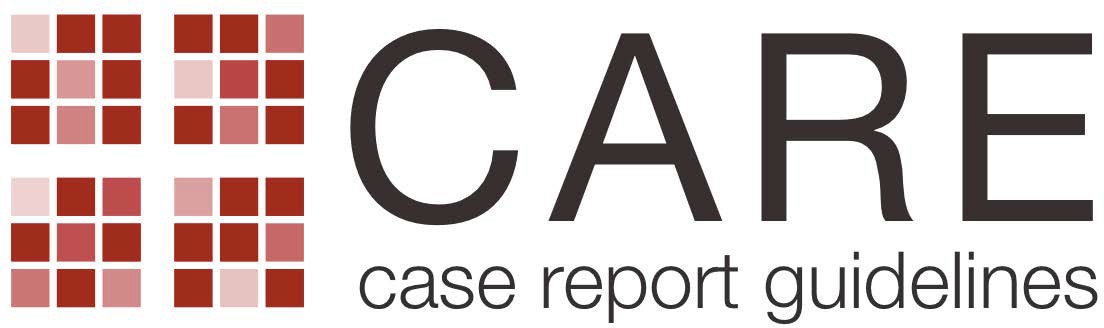
CARE Checklist of information to include when writing a case report
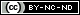


**Topic Item Checklist item description Reported on Line**

**Title 1** The diagnosis or intervention of primary focus followed by the words “case report” . . . . . . . . . . . . . . . . . . Lines 1−2

**Key Words 2** 2 to 5 key words that identify diagnoses or interventions in this case report, including "case report" Lines 51−52

**Abstract**

**(no references)**

**3a** Introduction: What is unique about this case and what does it add to the scientific literature? Lines 29−33

**3b** Main symptoms and/or important clinical findings . . . . . . . . . . . . . . . . . . . . . . . . . . . . . . . . . . . . . . . . . . . . . . . . . . . Lines 33−39

**3c** The main diagnoses, therapeutic interventions, and outcomes Lines 33−45

**3d** Conclusion—What is the main “take-away” lesson(s) from this case? Lines 45−49

**Introduction 4** One or two paragraphs summarizing why this case is unique (**may include references**) Lines 78−81

**Patient Information 5a** De-identified patient specific information Lines 84−146

**5b** Primary concerns and symptoms of the patient Lines 84−113

**5c** Medical, family, and psycho-social history including relevant genetic information Lines 84−113

**5d** Relevant past interventions with outcomes Lines 115−119

**Clinical Findings**

**Timeline**

**Diagnostic Assessment**

**Therapeutic Intervention**

**Follow-up and Outcomes**

1. Describe significant physical examination (PE) and important clinical findings Lines 87−113
2. Historical and current information from this episode of care organized as a timeline Lines 334−338

**8a** Diagnostic testing (such as PE, laboratory testing, imaging, surveys). Lines 84−103

**8b** Diagnostic challenges (such as access to testing, financial, or cultural) N/A

**8c** Diagnosis (including other diagnoses considered) Lines 83, 92−103

**8d** Prognosis (such as staging in oncology) where applicable N/A

**9a** Types of therapeutic intervention (such as pharmacologic, surgical, preventive, self-care) . . . . . . . . . . . . . . . . . Lines 90−91; 115−123

**9b** Administration of therapeutic intervention (such as dosage, strength, duration) Lines 121−123

**9c** Changes in therapeutic intervention (with rationale) N/A

**10a** Clinician and patient-assessed outcomes (if available) Lines 125−132

**10b** Important follow-up diagnostic and other test results Lines 134−143

**10c** Intervention adherence and tolerability (How was this assessed?) N/A

**10d** Adverse and unanticipated events Lines 145−146

**Discussion 11a** A scientific discussion of the strengths AND limitations associated with this case report Lines 152−176

**11b** Discussion of the relevant medical literature **with references** Lines 152−176

**11c** The scientific rationale for any conclusions (including assessment of possible causes) Lines 178−182

**11d** The primary “take-away” lessons of this case report (without references) in a one paragraph conclusion Lines 178−182

**Patient Perspective 12** The patient should share their perspective in one to two paragraphs on the treatment(s) they received . . . . N/A

**Informed Consent 13** Did the patient give informed consent? Please provide if requested . . . . . . . . . . . . . . . . . . . . . . . . . . . . . . . . . . . . . . **Yes** ✓ **No
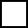
**
